# Motivations for Intravaginal Product Use among a Cohort of Women in Los Angeles

**DOI:** 10.1371/journal.pone.0151378

**Published:** 2016-03-11

**Authors:** Joelle M. Brown, Eugenie Poirot, Kristen L. Hess, Stephen Brown, Michele Vertucci, Marjan Hezareh

**Affiliations:** 1 Department of Epidemiology and Biostatistics, University of California San Francisco, San Francisco, California, United States of America; 2 Department of Epidemiology, University of California Los Angeles, Los Angeles, California, United States of America; 3 AIDS Research Alliance of America, Los Angeles, California, United States of America; Örebro University, SWEDEN

## Abstract

**Objective:**

Intravaginal practices—including behaviors such as intravaginal cleansing and insertion of products—have been linked to a number of adverse reproductive health outcomes, including increased risk for bacterial vaginosis, sexually transmitted infections, and HIV. Currently, little is known about the motivations for intravaginal practices among women in the United States. The objective of this study was to identify and describe motivations for intravaginal washing and intravaginal insertion of products among women of differing ages and racial/ethnic groups.

**Methods:**

Between 2008 and 2010, we enrolled a convenience sample of sexually active women aged 18–65 years living in Los Angeles recruited through community education and outreach activities in HIV/AIDS service organizations, women’s health clinics, community-based organizations, and HIV testing sites. At the enrollment visit, women completed a self-administered, computer-assisted questionnaire covering demographics, sexual behaviors, intravaginal practices, and motivations for intravaginal practices over the past month and past year.

**Results:**

We enrolled 141 women; 34% of participants were Caucasian, 40% African American, and 26% Latina. Peri-sexual intravaginal washing was common in all groups, whether to clean up after sex (70%) or to prepare for sex (54%). African American women were more likely to report learning to wash intravaginally from their mothers compared to Latina or Caucasian women (70% vs. 49%, *P* = 0.04). Sixty-one percent of African American women reported using a douching device over the past year compared to 41% of Latina and 40% of Caucasian women (p = 0.02). Younger women were more likely to report that their male partners wanted them to wash intravaginally than older women (77% vs. 24%, *P*<0.01), and more likely to report the removal of odors as a motive than older women (65% vs. 40%, *P* = 0.04). The most commonly used intravaginal products included sexual lubricants, petroleum jelly, body lotions, oils, and wet wipes. Use of these products varied by race, and motives given included increasing lubrication, preparing for sex, smelling good, and preventing sexually transmitted infections.

**Conclusion:**

Women’s intravaginal practices and motivations for these practices differ across race and age. Motivations for use also vary by type of intravaginal product used. Given that some intravaginal practices have been shown to be harmful, interventions, programs and counseling messages to encourage less harmful practices are needed, and should consider underlying motivations that influence women’s vaginal practices. Practitioners may use these results to better support women in achieving vaginal health.

## Introduction

Intravaginal practices have been linked to a number of adverse reproductive health outcomes, including increased risk for bacterial vaginosis (BV), sexually transmitted infections (STI), and HIV [1–4]. Intravaginal practices consist of a variety of behaviors, including wiping, cleansing, douching, or the insertion of over-the-counter products, such as sexual lubricants, into the vagina [5, 6]. These behaviors can disturb the normal protective vaginal environment, which is based in part on hydrogen peroxide, lactobacillus, and pH. Such practices can affect intravaginal mucosal integrity and increase one’s susceptibility to BV, STIs and HIV. For example, soaps used to wash the inside of the vagina can cause chemical damage and increase vaginal pH [7], contributing to the growth of organisms associated with BV [8]. BV is the most common cause of vaginitis in women of reproductive age [9], and a condition shown to increase women’s risk of STI acquisition, including HIV [10].

Research from both the developed and developing world has shown that intravaginal practices are common, and influenced by complex social, cultural, and biological factors [11]. However, the majority of research on the motivations for these practices in the United States has focused on douching, which is generally thought of as the use of a pressurized solution to cleanse intravaginally, usually with a douching device. Currently little is known about the motivations for other types of intravaginal practices, such as the insertion of products into the vagina, among women in the United States. Through understanding the motivations for intravaginal practices among women in different populations, the public health community can develop effective counseling messages and interventions to encourage less harmful practices, and better support women’s vaginal health.

In this paper we use data collected as part of a cohort study among women in the United States to contextualize the motivations for intravaginal washing and intravaginal insertion of products in a study population where data are lacking. The overall objectives of the cohort study were to (1) quantify the prevalence and frequency of vaginal and rectal practices and product use, (2) examine the associations between these practices and reproductive tract infections, and (3) describe the context and motivations for these practices among an ethnically diverse population. Results from objectives one and two have been published previously, where findings demonstrated an increased risk of BV associated with the intravaginal insertion of products [2]. The objective of this paper is to specifically address objective three and describe and compare the motivations for intravaginal washing and intravaginal insertion of products among women at the time of enrollment into the cohort, among African American, Latina, and Caucasian women. We hypothesized that intravaginal practices may differ by age, and across racial and ethnic groups. We report on those findings and discuss their implications for vaginal health.

## Methods

This prospective cohort study was implemented by the University of California, Los Angeles, and the AIDS Research Alliance of America in Los Angeles, California between 2008 and 2010. A convenience sample of sexually active women aged 18–65 years was selected through community education and outreach activities conducted by the AIDS Research Alliance staff in HIV/AIDS service organizations, women’s health clinics, community-based organizations, and HIV testing sites in Los Angeles. Women greater than 18 years of age were invited to attend screening visits at the AIDS Research Alliance clinic and, if eligible, and willing to attend study visits, enrolled. Women were excluded if they planned to move from the area in the next 12 months.

To achieve an ethnically diverse sample, comprising of Los Angeles’ three major racial groups, where women and girls of color make up more than 70% of the County’s female population [12], we enrolled near equal numbers of participants who self-identified as non-Hispanic Caucasian, non-Hispanic African American or black, or Hispanic or Latina. Women who self-identified as Asian were eligible; however, few showed up to the clinic for screening. In total, four Asian women were enrolled but given that there were too few women in these categories to warrant a separate stratum, these women (none of which identified as Latina) were included in the race category “Non-hispanic White”. We saw no evidence of an effect on estimates in sensitivity analyses using this choice of categorization. The sample size for evaluating differences in motivation by ethnicity was based on feasibility rather than specific power calculations. It was estimated that a sample of 40–50 women per ethnicity group would give us 80% power to identify moderate differences in reported motivations (>25%) between groups as statistically significant at the 0.05 level. Ethical approval for the study was obtained from the institutional review boards of the University of California, Los Angeles and the AIDS Research Alliance. All women provided informed consent prior to study participation. Participants were seen at the AIDS Research Alliance clinic at enrollment and then after 12 months. Data presented in this cross-sectional analysis are based only upon data obtained at enrollment. Additional details of the methods are described elsewhere [2].

At enrollment, a structured, self-administered, computer-assisted questionnaire was used to collect all survey responses (Sensus 4.2, Sawtooth Technologies, Northbrook, Illinois). Skip patterns, logic and range checks were programmed into the computer-assisted questionnaire to ensure high quality data. Data on demographic factors (e.g., age, race and ethnicity), sexual behaviors, intravaginal practices, and motivations for intravaginal practices was collected. Women were specifically asked about types of and reasons for intravaginal practices over the previous year and during the last month, as well as during the last act of vaginal intercourse. We also asked women who taught them to wash intravaginally. In addition, questions on partner characteristics, contraceptive use, and vaginal symptoms were asked but given the scope of this analysis, are not described. Counseling was offered to women by study staff to help them distinguish between intravaginal practices (beyond the introitus) and practices that involved the external genitalia (e.g., mons pubis, vulva). Our survey questions adopted the classification framework from the World Health Organization (WHO) Gender, Sexuality and Vaginal Practices study to define intravaginal practices [5] as either: 1) intravaginal washing, or 2) intravaginal insertion of products. Intravaginal washing included intravaginal cleaning on the inside of the vagina, beyond the introitus, with soap, commercially prepared solutions, or other household products, with or without douching devices. In contrast, the intravaginal insertion of products, comprised of the insertion of over-the-counter products (e.g., petroleum jelly, sexual lubricants, wet wipes) into the vagina, beyond the introitus, with or without an applicator. This typology is based on definitions of practices identified from an extensive, qualitative investigation and household surveys in four countries [6] that has become standard among researchers in the field. Each woman completed the questionnaire on her own in a private room at the study site, and study staff were available outside the room to respond to questions, if needed. The questionnaire was available in both English and Spanish.

Data were analyzed using STATA 12.1 (StataCorp, College Station, Texas). Sociodemographics and the prevalence and types of intravaginal practices were tabulated. Proportions were calculated for binary measures and measures of tendency of dispersion (median, interquartile range, range) were described for continuous variables. Estimates were calculated overall, by participant age, and by race and ethnicity. Univariable associations between participants’ motivations for intravaginal practices (i.e., washing and product use), age, race and ethnicity, and type of products used are presented. Univariate differences in categorical variables between study groups were assessed using two-sided Fisher’s exact tests. A non-parametric test for trend was performed across age when meaningful differences were found. Due to small numbers of women reporting use of specific intravaginal products for specific reasons formal statistical testing to evaluate the difference in motivations for each type of product used was not performed.

## Results

One hundred fifty women were screened and consented; of these 141 women were enrolled and completed the baseline questionnaire; nine women did not complete the baseline interview. Overall, data quality was high with no specific questions commonly left unanswered. There was no missing data for age, race, education, intravaginal practices, condom use, and sexual practices. Ninety-eight percent of the data on other variables were complete.

The median age of the women was 32 years (interquartile range 25–44, range 18–65 years). One third (34%) of women were Caucasian, 40% were African American, and 26% were Latina. Most of the women (87%) were born in the United States. The majority (89%) had completed at least a high school education. The median monthly income among women in this cohort was $750 [interquartile range $221–1000, range $0–4500]. Age, monthly income and education did not differ by race/ethnicity group (Table 1).

**Table 1:** Characteristics of participants measured at enrollment, by race/ethnicity.

		Race/Ethnicity			
	Total	Caucasian	African American	Latina	
	N = 141	N = 48	N = 56	N = 37	P-value
**Age**	32 [25–44]	33 [25–48]	31 [24–44]	35 [24–42]	0.55
**Born in the United States**	122 (86.5)	42 (87.5)	55 (98.2)	25 (67.6)	<0.01
**Monthly income (US$)**	750 [221–1000]	870 [221–1200]	600 [221–1000]	584 [210–900]	0.58
**Education**					
< High school	16 (11.4)	3 (6.3)	8 (14.3)	5 (13.5)	
High school graduate	46 (32.6)	13 (27.1)	19 (33.9)	14 (37.8)	
Some college	51 (36.2)	19 (39.6)	18 (32.1)	14 (37.8)	
College graduate	26 (18.4)	12 (25.0)	10 (17.9)	4 (10.8)	0.52
**Sexual History**					
Vaginal intercourse with a man (ever in lifetime)	134 (95.0)	44 (91.7)	53 (94.6)	37 (100.0)	0.44
Sex with a woman (ever in lifetime)	57 (40.4)	21 (43.8)	22 (39.3)	14 (37.8)	0.66
Sex with a woman (past mo.)	17 (12.1)	5 (10.4)	9 (16.1)	3 (8.1)	0.49
Sex with a man (past mo.)	118 (83.7)	38 (79.2)	46 (82.1)	34 (91.9)	0.46
Number of male sexual partners (past mo.)	1 [1–1]	1 [1–1]	1 [1–1]	1 [1–2]	0.29
Any unprotected vaginal sex (past mo.)	51 (36.2)	15 (31.3)	22 (39.3)	14 (37.8)	0.68
Frequency of vaginal sex (past mo.)	5 [2–12]	7 [4–15]	5 [1–10]	6 [2–12]	0.60
**Sexually Transmitted Infections diagnosed at Enrollment**					
HIV	38 (27.0)	13 (27.1)	15 (26.8)	10 (27.0)	0.99
HSV-2	73 (51.8)	20 (41.7)	34 (60.7)	19 (51.4)	0.17
*T*. *vaginalis*	5 (3.6)	2 (4.2)	2 (3.6)	1 (2.7)	1.0
*C*. *trachomatis*	1 (0.7)	0	1 (1.8)	0	1.0
*N*. *gonorrhoeae*	1 (0.7)	1 (2.1)	0	0	0.6

Data are n (%) or median [interquartile range]

When asked about sexual partners over the past month, most women (93%) reported a male sexual partner and 7% reported a female sexual partner. The median number of acts of vaginal intercourse with a man was 5 times over the past month (IQR 2–12), and one third (36%) of participants reported unprotected vaginal intercourse during this timeframe.

### Intravaginal washing

Nearly half (45%, 95% CI 36.3–53.3%) of women reported washing the inside of the vagina during the past month; 57% of women reported doing so in the last year (Table 2). Two-thirds of African American women reported intravaginal washing over the past year compared to half of Latina and Caucasian women (66% vs. 51%, *P* = 0.1). The majority of women (85%; 68/80) who reported intravaginal washing over the past year reported using a douching device in this practice. Overall, 61% of African American women reported using a douching device over the past year compared to 41% of Latina and 40% of Caucasian women (*P* = 0.02). Intravaginal washing was commonly reported in all age groups, though older women were somewhat more likely to report washing than younger women; two-thirds (65%) of women aged 34–65 years reported intravaginal washing over the past year compared to half (51%) of women aged 18–33 (*P* = 0.09). Younger women and older women tended to be equally likely to report using a douching device over the past year (*P* = 0.61) (Table 2). HIV positive women were more likely to report intravaginal washing over the past year than HIV negative women (68% vs. 52%, *P* = 0.07).

**Table 2:** Motivations for intravaginal washing over the past year by race/ethnicity and age.

		Race/Ethnicity				Age category (years)				
	Total	Caucasian	African American	Latina	P-value	18–25	26–33	34–44	45–65	P-value
	N = 141	N = 48	N = 56	N = 37		N = 43	N = 31	N = 34	N = 33	
**Any intravaginal washing, past year**	80 (57)	24 (50)	37 (66)	19 (51)	0.25	22 (51)	15 (48)	22 (65)	21 (64)	0.40
**Intravaginal washing with a douching device, past year**	68 (48)	19 (40)	34 (61)	15 (41)	0.02	18 (42)	14 (45)	19 (56)	17 (52)	0.61
Why did you wash intravaginally?*										
To be clean/fresh	67 (84)	19 (79)	30 (81)	18 (95)	0.40	19 (86)	13 (87)	18 (82)	17 (81)	1
To clean up after sex	56 (70)	16 (67)	25 (68)	15 (79)	0.70	15 (68)	13 (87)	15 (68)	13 (62)	0.51
To prepare for sex	43 (54)	13 (54)	20 (54)	10 (53)	0.98	12 (55)	10 (67)	10 (45)	11 (52)	0.65
To be sexy	25 (31)	6 (25)	14 (38)	5 (26)	0.45	6 (27)	8 (53)	5 (23)	6 (29)	0.23
To remove odor	41 (51)	12 (50)	20 (54)	9 (47)	0.83	13 (59)	11 (73)	7 (32)	10 (48)	0.08
To remove germs	38 (48)	13 (54)	18 (49)	7 (37)	0.50	11 (50)	10 (67)	7 (32)	10 (48)	0.22
Partner wanted you to	46 (58)	14 (58)	20 (54)	12 (63)	0.86	17 (77)	11 (73)	13 (59)	5 (24)	<0.01
Who taught you to wash intravaginally?*										
Mother	47 (59)	11 (46)	26 (70)	10 (53)	0.10	10 (45)	10 (67)	12 (55)	15 (71)	0.23
Father	1 (1)	0	1 (3)	0	1	1 (5)	0	0	0	1
Sister	6 (8)	1 (4)	3 (8)	2 (11)	0.76	2 (9)	1 (7)	2 (9)	1 (5)	1
Brother	1 (1)	1 (4)	0	0	0.54	1 (5)	0	0	0	1
Other Relative	5 (6)	0	5 (14)	0	0.07	2 (9)	1 (7)	1 (5)	1 (5)	1
Friend	7 (9)	4 (17)	2 (5)	1 (5)	0.35	1 (5)	1 (7)	3 (14)	2 (10)	0.77
Coworker	1 (1)	1 (4)	0	0	0.54	0	0	0	1 (5)	0.44
Partner	4 (5)	1(4)	1 (3)	2 (11)	0.45	1 (5)	0	1 (5)	2 (10)	0.77
Media	4 (5)	0	2 (5)	2 (11)	0.26	2 (9)	0	0	2 (10)	0.36
You taught yourself	43 (54)	13 (54)	17 (46)	13 (68)	0.32	15 (68)	7 (47)	12 (55)	9 (43)	0.43

Data are n (%); column totals add up to more than 100% as women were allowed to choose multiple answers

*Among those reporting intravaginal washing over the past year

Women reported several motivations for intravaginal washing. The most common motivations related to personal hygiene and sexuality. Overall, 84% of women reported cleaning the inside of their vagina during the past year to be clean/fresh (Table 2). Approximately, one-third of women (31%) reported that intravaginal washing was motivated by the desire to be sexy. Peri-sexual intravaginal washing was common, whether to clean up after sex (70%) or to prepare for sex (54%). These reported motivations did not differ significantly by age or ethnicity.

In contrast, over half of women (58%) reported that their partners encouraged intravaginal washing. Younger women were more likely to report being encouraged by their partners to wash intravaginally than older women (Table 2). Specifically, 77% of women aged 18–25 years, 73% of women aged 26–33 years, 59% of women aged 34–44 years, and 24% of women aged 45–65 years reported that they washed intravaginally because their partners wanted them to (*P*<0.01; nonparametric test for trend across ordered groups, *P* < 0.01). In addition, younger women (18–33 years) reported the removal of odors as a motivating factor more often than older women (34–65 years) (65% vs. 40%, *P* = 0.03).

Women reported learning to wash intravaginally from several sources, with the majority learning from their mothers (59%) or being self-taught (54%) (Table 2). African American women were more likely to report being taught by their mothers than Latina or Caucasian women (70% vs. 49%, *P* = 0.04). Few women reported learning to wash intravaginally from their sisters (8%), friends (9%), partners (5%), or the media (5%) (Table 2).

### Intravaginal insertion of products

Overall, 58% of women reported inserting some type of over-the-counter product (other than tampons) inside the vagina over the past month (Table 3). Over-the-counter products included petroleum jelly, oils, lotions, and wet wipes, and were used both in the context of sex and at other times. Overall, older women were more likely than younger women to report the use of intravaginal products over the past month (Table 3); two-thirds (64%) of women aged 26–65 years compared with 44% of women aged 18–25 years reported any intravaginal product use over the past month (*P* = 0.03).

**Table 3:** Intravaginal insertion of products over the last month, by race/ethnicity and age.

		Race/Ethnicity				Age category (years)				
	Total	Caucasian	African American	Latina	P-value	18–25	26–33	34–44	45–65	P-value
	N = 141	N = 48	N = 56	N = 36		N = 43	N = 31	N = 34	N = 33	
**Any intravaginal product use, past mo.**	82 (58)	31 (65)	27 (48)	24 (65)	0.15	19 (44)	20 (65)	22 (65)	21 (64)	0.17
Types of intravaginal products used, past mo.*										
Commercial sexual lubricant	60 (73)	26 (84)	15 (56)	19 (79)	0.04	12 (63)	15 (75)	16 (73)	17 (81)	0.67
Petroleum jelly	15 (18)	5 (16)	8 (30)	2 (8)	0.14	4 (21)	4 (20)	1 (5)	6 (29)	0.18
Lotion	17 (21)	4 (13)	6 (22)	7 (29)	0.32	6 (32)	1 (5)	7 (32)	3 (14)	0.08
Oils	11 (13)	2 (6)	6 (22)	3 (13)	0.23	1 (5)	1 (5)	5 (23)	4 (19)	0.23
Wet wipes	18 (22)	6 (19)	9 (33)	3 (13)	0.22	5 (26)	7 (35)	2 (9)	4 (19)	0.19

Data are n (%); column totals add up to more than 100% as women were allowed to choose multiple products

*Among those reporting insertion of any intravaginal product over the past month

Among women reporting the insertion of an intravaginal product (n = 82), the most commonly reported products were commercial sexual lubricants (73%), wet wipes (22%), body lotions (21%), petroleum jelly (18%) and oils (13%). Older women aged 34–65 years were more likely to report intravaginal use of oils than younger women (21% vs. 5%, *P* = 0.05). Younger women, aged 18–33 years, were more likely to report intravaginal use of wet wipes compared with older women aged 34–65 (32% vs. 14%, *P* = 0.06). African American women were more likely to report intravaginal use of petroleum jelly (30% vs. 13%, *P* = 0.06), wet wipes (33% vs. 17%, *P* = 0.10), and oils (22% vs. 9%, *P* = 0.10) compared with Caucasian and Latina women. In contrast, Caucasian and Latina women were more likely to report intravaginal use of a commercial sexual lubricant compared with African American women (82% vs. 56%, *P* = 0.01).

Most women reported inserting intravaginal products to increase lubrication, to increase sexual pleasure, to be ready for sex, and to reduce discomfort and dryness (Table 4). For example, among women who reported intravaginal use of a commercial sexual lubricant, the majority reported using it to increase lubrication (78%) and to increase sexual pleasure (53%). Similarly, the majority of women who reported intravaginal use of petroleum jelly reported using it to reduce dryness (69%) and reduce discomfort (50%). Women’s motivations behind the use of oils were mainly to increase lubrication (73%), to increase sexual pleasure (64%), to be ready for sex (55%), and to reduce vaginal dryness (55%) (Table 4).

**Table 4:** Motivations for intravaginal insertion of products during last vaginal intercourse, by type of product used.

	Commercial sexual lubricant	Oils	Petroleum jelly	Body lotions	Wet wipes
Reasons for use	N = 64	N = 11	N = 16	N = 19	N = 14
To be clean	2 (3)	1 (9)	1 (6)	2 (11)	13 (93)
To be ready for sex	38 (59)	6 (55)	6 (38)	9 (47)	5 (36)
To clean up after sex	2(3)	0	2 (13)	0	10 (71)
To increase lubrication	50 (78)	8 (73)	10 (63)	12 (63)	4 (29)
To increase sexual pleasure	34 (53)	7 (64)	4 (25)	6 (32)	2(14)
To reduce vaginal dryness	38 (59)	6 (55)	11 (69)	14 (74)	3 (21)
To reduce your discomfort	29 (45)	4 (36)	8 (50)	6 (32)	2 (14)
To smell good/reduce odor	7 (11)	4 (36)	2 (13)	6 (32)	6 (43)
To protect against STIs	3 (5)	0	3 (19)	1 (5)	1 (7)

Data are n (%); column totals add up to more than 100% as women were allowed to choose multiple reasons

Intravaginal use of body lotions was primarily influenced by a perceived need to reduce dryness (74%) and to increase lubrication (63%). The most common reasons for intravaginal use of wet wipes were to be clean (93%), to clean up after sex (71%) and to smell good (43%).

Finally, some women reported use of these products to protect from STIs. For instance, 19% of women who used petroleum jelly reported that they used it to protect from an STI the last time they had vaginal intercourse. In general, women’s reasons for using intravaginal products during last vaginal sex were consistent across age, race and ethnicity.

## Discussion

Intravaginal washing and insertion of products are common practices among African American, Caucasian, and Latina women across all age groups in this Los Angeles cohort. We found that nearly half of women report washing inside the vagina and over half of women report inserting some type of over-the-counter product (other than tampons) inside the vagina over the past month. Women’s intravaginal practices and their motivations for use differ across race and age categories, and motivations vary by type of intravaginal product used. While most women reported the use of commercial sexual lubricants, many also reported inserting petroleum jelly, lotion, oils, and wet wipes into the vagina to be ready for, or to increase the pleasure of vaginal sex. African American women were more likely to report use of products not designed for and potentially harmful upon insertion into the vagina, such as petroleum jelly, oils, and wet wipes. We found that most women wash intravaginally because they feel it is necessary for good hygiene. This finding is consistent with the findings of prior research [13–16]. Women also wash intravaginally to prepare for sex, and to clean up after sex, which also comports with previous studies [17]. Our study also found that half of women, particularly younger women, perceive that their male sexual partners want them to wash intravaginally, demonstrating the important influence of sexual partners on women’s intravaginal practices in this cohort.

Prior research has found that Caucasian women learn intravaginal practices from electronic and print media [18]. In contrast, we found that few women learn these practices from the media. Instead, a significant proportion of women of all ages report learning to wash intravaginally from their mothers. African American women, in particular, are more likely to report learning to wash intravaginally from their mothers. Two studies among adult women living in the southeastern United States similarly reported that mothers were the most common source of encouragement about douching among African American women [13, 18].

Most clinicians and the American College of Obstetricians and Gynecologists strongly recommend that women not wash or douche intravaginally [19]. Yet, in our study, approximately half of women aged 18–65 years report intravaginal washing over the past month. Our finding that intravaginal washing is reported by between one half and two-thirds of African American women are supported by other published estimates in the U.S. [20, 21]. For example, the National Survey of Family Growth (NSFG) in the U.S. estimates that 32% of women aged 15–49 years practice douching, and over half (58.5%) of African American women report douching over the past year [20]. The somewhat higher overall prevalence of intravaginal washing in our study (45% vs. 32% in NSFG) could be related to the composition of the specific populations studied (we ensured one-third of the study population was African American vs. 8% of the cohort being African American in NSFG), the older age range of our study population (18–65 years vs. 14–49 years in the NSFG), the more recent time frame of our study (2010 vs. 2002 in NSFG), the way in which the interview was conducted (self-reported, private, computer-assisted interviews vs. in-person interviews in NSFG) or the way in which we asked about intravaginal washing. In our study, the definition of intravaginal washing was modeled on the WHO Gender Sexuality, and Vaginal Practices Study group classification for vaginal practices [5], and defined as any insertion of a liquid into the vagina, beyond the introitus, with or without a douching device. In the NSFG, respondents were asked ‘“During the past 12 months, how often, if at all, did you douche?”. It is possible that women do not equate all forms of intravaginal washing with douching, which is generally thought of as the use of a pressurized solution to cleanse intravaginally, usually with a douching device [22], and this could have resulted in underreporting of intravaginal washing in the NSFG. These different definitions could account for some of the differences in prevalence, and also highlights the importance of using, where possible, definitions of intravaginal practices, products, and frequency that allow comparisons across populations and studies [22].

The strengths of this study lie in its design as an in-depth study of a variety of intravaginal practices including but not limited to douching in the United States. The comprehensiveness of the questionnaire allowed for a detailed evaluation of intravaginal practices among a cohort of women of diverse ages and ethnicities. The age and racial distribution of women who participated allowed us to examine differences between groups. The use of a self-administered questionnaire rather than an interviewer-administered questionnaire may have limited reporting bias of sensitive or stigmatized behaviors.

Limitations of this study were mainly due to its relatively small sample and the small numbers of women reporting certain types of intravaginal practices, which limited our ability to detect statistically significant associations. Furthermore, because of the sample selection and small sample size, results may not be generalizable to other groups. A purposive sample of women was chosen for this study not to be representative but rather to achieve balance across three major racial groups that comprise the majority of Los Angeles’ female population; Latinas make up the majority of the female population (47%), followed by white (28%), Asian Americans (14%) and African Americans (9%). However, a similar proportion of our sample completed at least a high school education (89%) compared to the approximately 84% of women in Los Angeles who have done so [12]. Thus, we recognize that these findings may not reflect the population as a whole. Nonetheless, given the lack of published findings on intravaginal practices among women in the United States, our study is a valuable contribution to the literature that describes the frequency, distribution, and variability associated with intravaginal practices among an ethnically diverse population of women.

Understanding women’s motivations for intravaginal practices is important because there is a growing body of international evidence suggesting that some intravaginal practices are harmful to women’s health. For example, intravaginal washing is not beneficial for vaginal hygiene, and may be associated with increased risk for BV, pelvic inflammatory disease, and STIs, including HIV [1, 2]. A meta-analysis among more than 10,000 HIV-negative women in sub-Saharan Africa found that intravaginal use of drying agents were associated with an increased risk of BV and HIV [1]. Numerous studies in developed countries have found associations between intravaginal practices and prevalent vaginal infections [16, 23] including our own study that found that women in this cohort from Los Angeles who reported intravaginal use of petroleum jelly were at increased risk for BV, taking into account vaginal symptoms, age, race, HIV status, and sexual activity [2]. Little is known about the possible effects of intravaginal product use on uptake or effectiveness of other HIV/STI prevention technologies. Because intravaginal practices appear to increase the risk of acquiring BV and HIV, the population-attributable risks and related costs could be large, especially in settings and populations in which these practices are most prevalent [1].

This study illustrates that personal, sexual, relational, and social factors influence women’s intravaginal practices and that these factors vary by race and age, and type of product. Additional, larger studies and in-depth qualitative analyses to further understand these important influences is essential for the development of interventions and counseling messages to improve vaginal health. Evolving evidence supports a multi-pronged approach to educating women and men about these common, and potentially deleterious intravaginal practices. First, we should develop appropriate public health messages to caution against harmful intravaginal practices. A pilot study among HIV-infected women in Zambia suggested that a behavioral intervention, involving an individual, interactive socio-educational session about intravaginal practices, could decrease these behaviors; communication with sexual partners regarding intravaginal practices was higher for women receiving the intervention [24]. Educational messages targeted toward or possibly delivered by influential sources of information, such as parents or male partners should also be considered. It will also be important to critically assess the regulation, testing, and labeling of over-the-counter products that are used intravaginally. In clinical settings, the link between intravaginal practices and poor reproductive health outcomes can be addressed by clinicians whenever a woman tests positive for pregnancy, BV, candidiasis, or an STI, by asking her about her vaginal practices [25]. The detection of intravaginal practices could also be initiated by women’s healthcare providers at family planning visits or through preventive counseling to prevent adolescent and young adult women from engaging in unsafe behaviors. With careful consideration of the underlying motivations that influence women’s intravaginal practices, which vary across race, age, and product of choice, practitioners may develop effective counseling messages, interventions, and programs to better support women in choosing less harmful practices, and achieving vaginal health.
